# A Principal Neighborhood Aggregation-Based Graph Convolutional Network for Pneumonia Detection

**DOI:** 10.3390/s22083049

**Published:** 2022-04-15

**Authors:** Akram Ali Ali Guail, Gui Jinsong, Babatounde Moctard Oloulade, Raeed Al-Sabri

**Affiliations:** School of Computer Science and Engineering, Central South University, Changsha 410083, China; akramjuail@csu.edu.cn (A.A.A.G.); oloulademoctard@csu.edu.cn (B.M.O.); alsabriraeed@tu.edu.ye (R.A.-S.)

**Keywords:** pneumonia detection, transfer learning, convolution neural network, graph neural network, principal neighborhood aggregation

## Abstract

Pneumonia is one of the main causes of child mortality in the world and has been reported by the World Health Organization (WHO) to be the cause of one-third of child deaths in India. Designing an automated classification system to detect pneumonia has become a worthwhile research topic. Numerous deep learning models have attempted to detect pneumonia by applying convolutional neural networks (CNNs) to X-ray radiographs, as they are essentially images and have achieved great performances. However, they failed to capture higher-order feature information of all objects based on the X-ray images because the topology of the X-ray images’ dimensions does not always come with some spatially regular locality properties, which makes defining a spatial kernel filter in X-ray images non-trivial. This paper proposes a principal neighborhood aggregation-based graph convolutional network (PNA-GCN) for pneumonia detection. In PNA-GCN, we propose a new graph-based feature construction utilizing the transfer learning technique to extract features and then construct the graph from images. Then, we propose a graph convolutional network with principal neighborhood aggregation. We integrate multiple aggregation functions in a single layer with degree-scalers to capture more effective information in a single layer to exploit the underlying properties of the graph structure. The experimental results show that PNA-GCN can perform best in the pneumonia detection task on a real-world dataset against the state-of-the-art baseline methods.

## 1. Introduction

**Figure 1:**
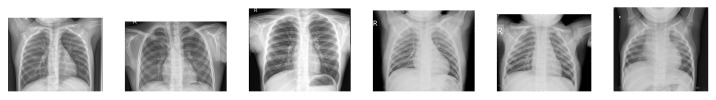
Examples of chest X-ray images of healthy people.

**Figure 2:**
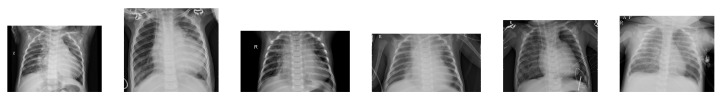
Examples of chest X-ray images of people with pneumonia presenting a bandlike opaqueness with a peripheral, mid-to-lower lung zone distribution—white infiltrates, a hallmark of pneumonia.

Pneumonia is an infection of the lower respiratory tract. It is caused by several pathogens, mainly viruses or bacteria. Pneumonia is more common in underdeveloped and developing countries, where overcrowding, pollution, and unsanitary environmental states make the situation more menacing, and medical resources are limited. Moreover, it is one of the leading causes of child mortality worldwide. It has been reported by the World Health Organization (WHO) to be the cause of one-third of child deaths in India [1]. Pneumonia is characterized by the presence of an abnormal area compared to the surrounding tissues in an X-ray image (see Figure 1 and Figure 2 for examples). Early diagnosis and treatment are critical in preventing further fatalities due to pneumonia. Analysis of the lungs by computed tomography (CT), magnetic resonance imaging (MRI), or X-rays is used for the diagnosis of pneumonia. Chest X-rays are commonly used to detect malignancy in the lungs, mostly because X-ray analysis constitutes is a relatively inexpensive and non-invasive lung exam [2]. However, as stated in [3], slight dissimilarities in terms of shape, scale, textures, and intensity, can complicate X-ray-based pneumonia detection, especially for patients below five years old. Other illnesses, such as congestive heart failure and lung scarring, could also be misclassified as pneumonia [1]. Thus, pneumonia detection requires an expert to use additional patient information to detect pneumonia symptoms from chest X-ray radiography, which is time-consuming for the radiologist, costly to hospitals, and not necessarily affordable for the users in need. Thus, designing an automated classification system to aid in detecting pneumonia has become a valuable research topic.

In the last few decades, researchers and medical practitioners have investigated the possibility of using deep learning in automated disease diagnosis systems. Deep learning techniques utilize the activation states of neurons to gradually assemble low-level features and automatically acquire higher-order abstract representations, avoiding complex feature engineering. Many deep learning models attempt to detect pneumonia by applying convolutional neural networks (CNNs) to X-ray radiographs, as they are essentially images [4]. The convolutional network approach involves hidden convolution and pooling layers to determinate spatially localized attributes through a set of receptive fields in kernel form. The convolution operator, also known as the feature detector of a CNN, is a filter in which the kernel filters input data, and the pooling operator is used to condense the resolution of each attribute map in the spatial dimensions, keeping the sizes of the attribute maps unchanged. Thus, CNNs are a class of neural networks with architecture that can achieve mappings between spatially distributed grid data of random lengths, making it flawless for classifying images. The standard CNN model pipeline consists of an input layer, a set of convolution layers, optional pooling layers with a fully connected neural network, and a final layer. The convolutional neural networks applied for pneumonia detection include VGGNet, Inception, ResNet, DenseNet, and AlexNet. Although these convolution-based approaches have achieved great performances in pneumonia detection to some extent, they can only achieve the higher-order features of ground objects in a distribution region, and fail to adaptively catch the geometric changes of different object regions in the X-ray images. Meanwhile, the boundary pixel classification attribute information may be mislaid while extracting the features, impacting the overall prediction. Hence, they cannot capture the higher-order feature information of all objects based on X-ray images. This is because the topology of the X-ray image’s dimensions does not always come with some spatially regular locality properties, which makes defining a spatial kernel filter for X-ray images non-trivial [5]. One solution to this limitation is to use graph signal computation-based convolutions.

In the last couple of years, graph neural networks (GNNs) have become increasingly important and have been a very popular topic for both academia and industry in the processing of non-Euclidean data because of their ability to learn useful features from a graph. GNN can operate directly on the graph structure and aggregate and transfer neighborhood information [6]. At the same time, it can also grasp higher-order representations of irregularly distributed data. Advances in computer vision show that Graph Neural Network (GNN) architectures perform very well in several image-processing tasks, such as object detection, segmentation, classification, and medical image computing. Shen et al. [7] improved the performance accuracy by applying the graph convolutional neural network to hyper-spectral remote sensing image classification. Cai et al. [8] combined graph convolution integration and a cross-attention mechanism for classification of remote sensing images. They first obtained low-dimensional attributes, which are more expressive. Next, they performed the prediction on the hyperspectral data using attributes and the relationship between them, which were generated by the graph convolution integration algorithm. Although these methods effectively learn pixel distribution in an image, they cannot capture enough information from the neighbors of the node in a single layer, leading to limitations in their expressive power and learning ability.

In this paper, we propose an efficient principal neighborhood aggregation-based graph convolutional network framework for pneumonia detection (PNA-GCN). Our proposed model has three essential components: feature extraction, graph feature reconstruction, and representation learning. Unlike typical ML approaches, which are heavily based on manually-crafted features, our model extracts abstract data features in two main steps: transferring state-of-the-art CNNs to extract features, and graph reconstruction based on the extracted features. In our method, we utilize deep-learning based algorithms that are effective at generalization. Specifically, we achieve feature extraction with the use of a transfer learning approach. After extracting features using a trained CNNs network, we build the features graph, in which each feature extracted from each image is represented as a node of the graphs. Finally, we propose a GCN with principal neighborhood aggregation, which combines multiple aggregators with degree-scalers.

To sum up, the key contributions of the work are summarized as follows:

1. To the best of our knowledge, we made the first attempt to use the graph convolutional network approach for pneumonia detection.
2. We propose PNA-GCN, an efficient principal neighborhood aggregation-based graph convolutional network framework for pneumonia detection. In PNA-GCN, we propose a new graph-based feature construction utilizing the transfer learning technique to extract features and construct the graph from images. Then, we propose a principal neighborhood aggregation-based graph convolutional network, in which we integrate multiple aggregation functions in a single layer with degree-scalers to enable each node to gain a better understanding of the distribution of messages it receives.
3. The performance of the proposed method is evaluated on the publicly available chest X-ray datasets. The accuracy, precision, recall, and F1 score have been utilized to evaluate the effectiveness of the proposed method compared to existing work in the literature.

## 2. Related Work

Pneumonia has become increasingly popular as a research topic in recent years. Making a very accurate diagnosis and identifying the source of the symptoms in a timely manner is a big challenge for doctors in order to alleviate the suffering of their patients. As a result, when it comes to the analysis and processing of biomedical images, image processing and deep learning algorithms have produced quite good results [9,10,11]. Several significant additions to the current literature are reviewed in this section.

Recent advancements and the availability of massive datasets have enabled algorithms to outperform medical experts in a wide variety of biomedical tasks. For example, several biomedical image detection methods have been proposed utilizing deep learning algorithms. The challenges of biomedical image processing are discussed by [9]. Deep-learning-based approaches have been extensively used to detect several diseases. The authors of [10,12] proposed deep learning models for dermatologist-level classification of the skin cancer. Reference [13] proposed a method to depict the prostrate in MRI volumes utilizing a convolutional neural network (CNN). In [14], deep learning techniques were used to detect brain hemorrhaging in CT scans, along with a technique to detect diabetic retinopathy in the photographs of retinal fundus [15]. In [16], deep learning techniques are proposed for chest pathology detection. Several examination techniques have been used to examine disease detection by utilizing X-ray images [17,18,19]. An algorithm for the evaluation of scanning the line optimization is applied on a chest X-ray image to avoid diagnostic errors by eliminating all the other body parts [20]. The authors of [21] proposed CMixNet, a two-deep, three-dimensional customized mixed link network to classify and detect lung nodules. An approach that combines DenseNet and long-short term memory networks (LSTM) is proposed to exploit the abnormality dependencies [22].

Several works have been proposed methods on pneumonia classification. The authors of [23] have used EMD (earth mover’s distance) to classify infected and normal non-infected pneumonia lungs. The authors of [11,24] utilized a CNN model for pneumonia detection. Reference [25] discussed the performance of a customized CNN in detecting pneumonia and also differentiating between bacterial and viral types via pediatric CXRs. The region-based CNNs have been used to segment pulmonary images by utilizing the image augmentation for pneumonia detection [26]. AlexNet and GoogLeNet neural networks have been used with data augmentation without any pretraining [27]. A deep CNN model CheXNeXt with 121 layers was used by [28] to classify 14 different pathologies, including pneumonia, in frontial-view chest X-rays. To identify 14 thoracic diseases, researchers in [29] employed a localization strategy based on pre-trained DenseNet-121, as well as feature extraction, to identify the diseases. Deep-learning-based pneumonia classification algorithms were utilized by [30,31,32] to classify pneumonia. On the basis of chest computed tomography (CT) images, reference [33] introduced a new multi-scale heterogeneous three-dimensional (3D) convolutional neural network (MSH-CNN), which they called the MSH-CNN. For the diagnosis of pneumonia, the authors of [34] used a hierarchical convolutional neural network (CNN) structure and a unique loss function, sin-loss. The authors of [35] used Mask-RCNN, which utilized both global and local features for pulmonary image segmentation, with dropout and L2 regularization, along with dropout and L2 regularization. Using a 3D deep CNN (3D DCNN), Jung and colleagues [36] were able to create shortcut links. According to [37], they merged the outputs of several neural networks and arrived at the final forecast by utilizing a majority voting procedure. The results showed that the deep features were strong and consistent in detecting pneumonia.

More recently, Liang et al. [38] combined a 3D convolutional neural network (3D-CNN) and GCN to diagnose COVID-19 pneumonia. They used the 3D-CNN for extracting features from an initial 3D-CT images, and used these features to design a COVID-19 graph in GCN. Although their approach seems to be similar to ours, they have a slight difference. Indeed, their method requires three pieces of information, including equipment type, hospital information, and disease training sample label, which are not always all available in real word cases. Keicher et al. [39] proposed a holistic graph-based approach combining both imaging and non-imaging information. The study in [40] offers a unique semantic-interactive graph convolutional network (SIGCN) capable of leveraging topological information gained from knowledge networks to improve multilabel recognition performance. In summary, given that this is essentially an image classification problem, it is evident that pre-existing or innovative CNN models are used as classifiers. However, CNN has several disadvantages, such as over-fitting when the dataset includes class imbalance. In contrast, graph neural network (GNN)-based models can address issues such as over-fitting and class imbalance by using a graph neural network. Based on the experimental results obtained in various disciplines, it is clear that a GNN-based model works fast in general [41]. GNN, a relatively recent approach in the domain of deep learning, is used to solve graph classification challenges. As a result, GNN requires input data in the form of graph data. Considering all the advantages and novelties of GNN approach, we propose a GNN-based model to solve the problem of pneumonia detection.

## 3. Materials and Methods

**Figure 3:**
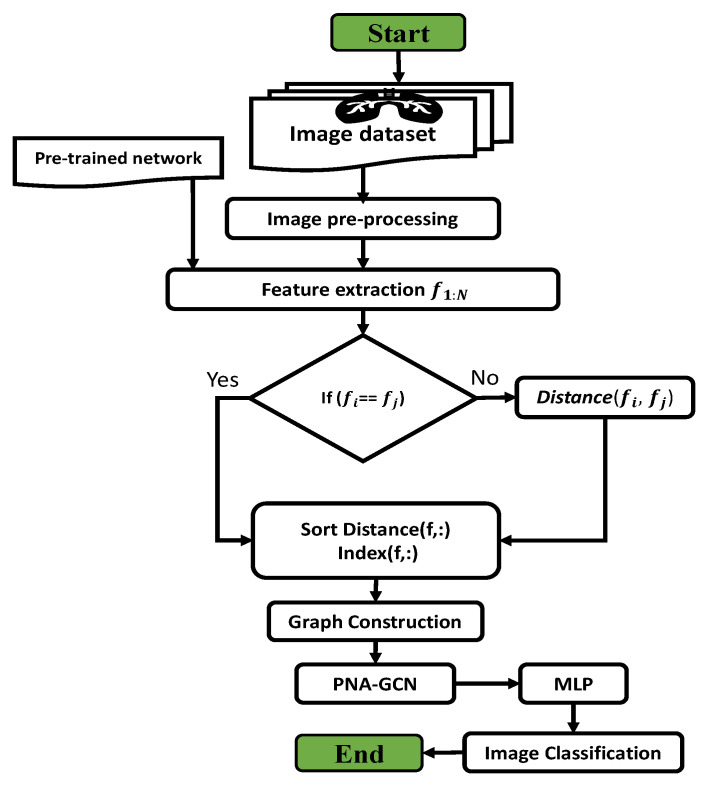
The methodology flowchart.

In this section, we describe the framework of the proposed model, as shown in Figure 3. In classical machine learning (ML) methods, data features are first extracted and then classified by classifiers. Our proposed PNA-GCN model has four essential components: data augmentation and preprocessing, feature extraction, graph feature reconstruction, and representation learning. We first apply the image preprocessing and data augmentation strategy on a pneumonia dataset. Unlike typical ML approaches which heavily based on manually-crafted features, our model extracts abstract data features utilizing two main steps: transferring state-of-the-art CNNs to extract features, and graph reconstruction based on the extracted features. In the following, we discuss the four main components of our proposed method for pneumonia disease detection.

### 3.1. Data Augmentation and Preprocessing

We used the data augmentation strategies that have been published in the literature to alleviate the problem of overfitting and improve the model’s capacity to generalize during the training phase, and to raise the amount and quality of the data [42]. The parameters that were used in data augmentation are listed in Table 1 on the right. The rescaling of the image is the first step in the process (reduction or magnification during the augmentation process). Following that, we perform rotation of the images, which is rotated at random throughout the training. It is the width shift that determines how far the images are offset horizontally, and it is the height shift that determines how far the images are offset vertically. In our situation, the width shift and the height shift were both 10 percent. Finally, the images were rotated horizontally and zoomed by a factor of 20 percent at random.

### 3.2. Feature Extraction

Feature extraction plays an important role in classification tasks, which affects the overall performance of classifiers. In our method, we utilized deep-learning-based algorithms that are effective at generalization. Specifically, we achieved feature extraction with the use of a transfer learning approach. In the feature extraction step, we first transferred state-of-the-art networks to the binary classification problem by replacing the top layers with new ones. After training on the training set, CNNs can generate preliminary findings and features. Generally, CNNs are trained using ImageNet [43], which provides classification results for 1000 categories. We used the general architecture of CNNs [44]. After transferring the state-of-the-art networks, we chose the CNN that performed the best on the test set to serve as a feature extractor for PNA-GCN.

In our proposed architecture, the transferring process was done by removing the top layers of the general architecture of CNNs and replacing them with dropout, transitional fully-connected (256 channels), and classification layers.

**Figure 4:**
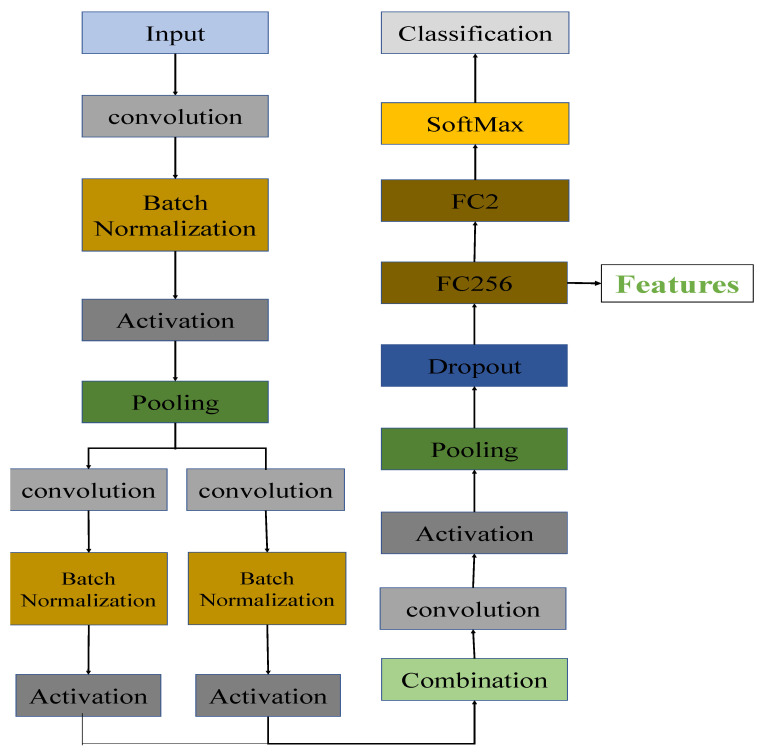
The architecture of the transferred CNNs. FC256 and FC2 are the fully connected layers with 256 and 2 channels, respectively.

Figure 4 shows the architecture of the transferred state-of-the-art CNNs after removing the top layers of CNNs and adding the new layers. After the final pooling layer, we added one dropout layer, one 256-channel transitional fully-connected layer, and a final 2-channel fully-connected layer for classification. In order to avoid overfitting problems during the training period, a dropout layer was included. If the size of a feature drops rapidly, the information contained within the feature will be significantly reduced. A transitional fully-connected layer is therefore on top of the dropout layer in order to prevent significant information loss from occurring. FC256 and FC2 are two fully-connected layers with 256 and 2 channels, respectively. As illustrated in Figure 4, the link between the final pooling layer and the softmax layer has been replaced with a connection between the last pooling layer and the newly added dropout layer. The parameters inside the CNNs were fine-tuned to offer better representations of the dataset after training with the pneumonia dataset for a limited number of epochs.

The details of how features are acquired in our architecture can be seen in the following steps: network transferring and feature extraction, where the acquired features were analyzed for underlying graph representation.

- In network transferring step, we first load a pre-trained convolution neural networks, which has been trained on the ImageNet dataset. Then, we remove the softmax and classification layers. After that, we add new layers, including a dropout layer and fully-connected layers with 256 and 2 channels (FC2 and FC256 in Figure 4). Using predefined parameters, we train the new networks on the training set of pneumonia dataset and save the networks and parameters.
- In feature extraction step, we first load the trained networks in the first step. Then, the target dataset is used as input to the network for feature extraction. We extract the features generated by the fully-connected layer (FC256 in Figure 4).

### 3.3. Graph Construction

**Figure 5:**
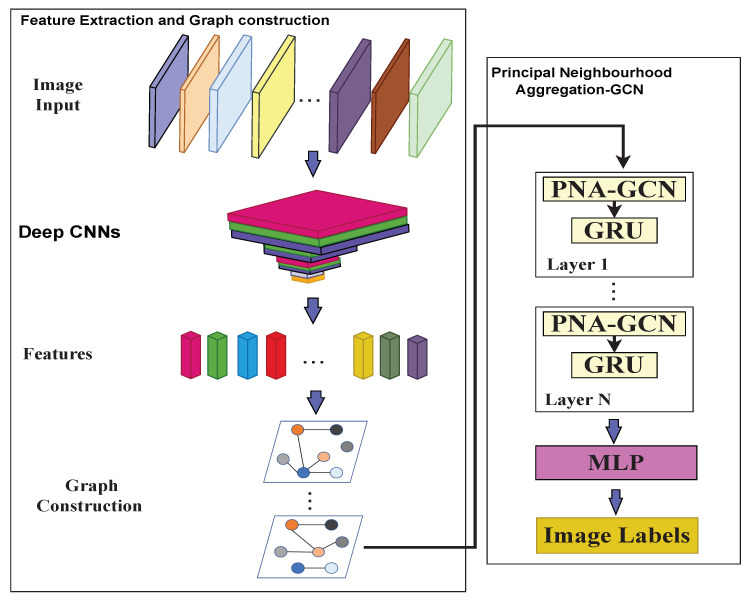
The overall architecture of the proposed model. In the architecture, we first use deep CNNs to extract the image vector features. Then, we construct graphs by calculating the distances between image vector features. Next is multiple principal neighborhood aggregation GCN. Each PNA-GCN is followed by a GRU layer. Finally, a MLP is used to classify features and images are labeled.

As shown in the overall architecture of the proposed model in Figure 5, first, the images were used as input to the pretrained deep CNNs described in in Section 3.2 to extract the image vector features. After extracting image vector features using trained deep CNNs network, we built the features graph, in which each feature extracted from an image is represented as a node of the graph, and the edges were built by calculating the distances between vector features, as described below. After that, the proposed principal neighborhood aggregation GCN was applied to the constructed graph. Finally, we applied the multi-layer perceptron MLP to pneumonia detection.

For faster computation, features are broken up into batches. Given the features $F\in R^{D\times M}$, where *D* is the number of images and *M* is the feature dimension, let the batch size be *B*. Then the number of batches *n* can be defined as:

(1) $$n=\left\lceil D/B\right\rceil$$

where $\left\lceil \cdot \right\rceil$ is the ceiling operation.

For each batch $F_{i}$, the graph $\left(V_{i},E_{i}\right)$ is constructed, which represents the underlying relationship between image vector features (nodes). $V_{i}$ indicates the nodes of the graph, which represents the feature vector for an image *i* in the batch, and $E_{i}$ represents the edges between nodes (image feature vectors). We utilize Euclidean distance to construct the edges among nodes. We build the edges between each node and its *k* neighbors if the *k* smallest Euclidean distance is found. Then, we build the adjacency matrix $A\in R^{B\times B}$. When the node $f_{i}$ and its neighbor $f_{i+1}$ are related, the value in $A_{i}$ at the position i,i+1 is set to a positive number.

Given a batch of features, wherein each feature represents an image, we construct the graph according to the following process:

First, we initialize the adjacency, distance, and index matrices as follows:

(2) $$A_{i}=zeros\left(B,B\right)$$

(3) Distance=Infinity(B,B)

(4) Sorted_Distance=zeros(B,B)

(5) Index=zeros(B,B)

where *B* is the number of samples in the batch. Note that $A_{i}$, Distance, Sorted_Distance, and Index are initialized variables.

The Distance is calculated as follows:

(6) $$Distance\left(j,m\right)=\sqrt[{}]{\sum_{h=1}^{M}{\left(f_{j}\left(h,1\right)-f_{m}\left(h,1\right)\right)}^{2}}$$

After calculating $A_{i}$, each feature $f_{j}$ in batch $F_{i}$ is recomputed as follows:

(7) $$f_{j}={\tilde{A}}_{i}^{j}F_{i}$$

where ${\tilde{A}}_{i}$ is the normalized adjacency matrix $A_{i}$ and ${\tilde{A}}_{i}^{j}$ is the jth row of ${\tilde{A}}_{i}$.

To build the graph $G_{i}$ for each batch of features $F_{i}$, we follow the following steps:

- Calculate the distances between each feature and the other features in the batch. Then, the distance matrix $Distance\in R^{B\times B}$ is computed.
- Sort Each row of the distance matrix in a ascending order.
- Generate the corresponding index matrix $Index\in R^{B\times B}$, in which the nearest *k* features are recorded in the batch $F_{i}$.
- Set the value of $A_{i}$ at the position (j,j) to 1 if the features $B_{i}$ and $B_{j}$ are nearest to each other based on the distance matrix.

### 3.4. Principal Neighborhood Aggregation-Based Graph Convolutional Network

In this section, first, we introduce GCN, and then we introduce the proposed principal neighborhood aggregation, which combines multiple aggregators with degree-scalers.

#### 3.4.1. Graph Convolutional Networks

GNNs are used to transfer a non-linear mapping g from a graph to a feature vector for the graph classification task.

(8) $$g:G\mapsto F_{G},$$

where $F_{G}$ is a feature vector of the entire graph *G* that is used for estimating the label of the graph. Based on the neighborhood aggregating techniques, a new perspective divides GNNs into two groups [45]. The spectral-based convolutional GNNs [46,47] are the first group (spectral GNN). The spectral decomposition of graphs inspired this group of GNNs, which try to approximate the spectral filters in each aggregating layer [48,49]. The spatial-based convolutional GNNs are the other type of GNN (spatial GNN). They do not set out to learn spectral properties of graphs; instead, they execute neighborhood aggregation based on the spatial relationships between nodes. The message passing neural network is a well-known example of a spatial GNN (MPNN) [50], and the GIN is another [51].

Inspired by CNNs, the GCN [49] is a multi-layer neural network that works on a graph and tries to find high-level features by combining information from the neighborhoods of graph nodes with information from other graph nodes. Formally, the undirected graph G=(V;E) is defined in GCN as the set of nodes and edges. In G, the adjacency matrix (A) is used to show the presence of an edge between each pair of nodes. Specifically, the spatial domain convolution operation may be used to implement the first-order approximation of the Chebyshev expansion of the spectral convolution operation:

(9) $$X^{\left(l\right)}=\sigma \left(\begin{array}{c} {\tilde{D}}^{-\frac{1}{2}}\tilde{A}{\tilde{D}}^{-\frac{1}{2}}{\tilde{X}}^{\left(l-1\right)}{\tilde{W}}^{\left(l\right)} \end{array}\right)\in R^{N\times C^{\left(l\right)}}$$

where $\tilde{A}=A+I$ is the adjacency matrix with the recurring loop, $\tilde{D}$ is the degree matrix, and ${\tilde{X}}^{\left(l-1\right)}$ is calculated as follows:

(10) $$X^{\left(l\right)}=\left[\begin{array}{ccc} x_{1}^{\left(l\right)} & \cdots & x_{C^{\left(l\right)}}^{\left(l\right)} \end{array}\right]\in R^{N\times C^{\left(l\right)}}$$

where $C^{\left(l\right)}$ represents the channel signals at the lth layer. This means that GCN implements the node feature with its neighbors via a layer-specific learnable weight matrix $W^{\left(l\right)}$ and non-linearity σ.

In contrast to spectral GNNs, spatial-based approaches define graph convolutions based on the spatial relations of a node. In general, this operation consists of the AGGREGATE and COMBINE functions:

(11) $$a_{v}^{\left(l\right)}=AGGREGATE^{\left(l\right)}\left(\begin{array}{c} \left\{\begin{array}{c} p_{v}^{\left(l-1\right)}:u\in N\left(v\right) \end{array}\right\} \end{array}\right),$$

(12) $$p_{v}^{\left(l\right)}=COMBINE^{\left(l\right)}\left(\begin{array}{c} p_{v}^{\left(l-1\right)},a_{v}^{\left(l\right)} \end{array}\right),$$

where $p_{v}^{\left(l\right)}\in R^{C^{\left(l\right)}}$ is the l−th layer feature at the v−th node. This means that the AGGREGATE function collects features from nearby nodes to produce an aggregated feature vector for the layer *l*, and the COMBINE function then combines the previous node feature $p_{v}^{\left(l-1\right)}$ with the aggregated node features $a_{v}^{\left(l\right)}$ to produce the current layer’s node feature $p_{v}^{\left(l\right)}$. The mapping is defined after this spatial process by:

(13) $$P_{G}=READOUT\left(\begin{array}{c} \left\{\begin{array}{c} p_{v}^{\left(l\right)}|v\in G \end{array}\right\} \end{array}\right)$$

#### 3.4.2. Principal Neighborhood Aggregation

Most work in the literature uses only a single aggregation method (Equation (11)) Mean, sum, and max aggregators are the most used in the state-of-the-art models. However, in the literature, we observed how various aggregators fail to discriminate between different messages when using a single GNN layer. In our method, we first apply degree-based scalers [52]. We use the logarithmic scaler $S_{amp}$, which is computed as follows:

(14) $$S_{amp}\left(d\right)=\frac{log(d+1)}{\delta },\delta =\frac{1}{\left|train\right|}\sum_{i\in train}log\left(d_{i}+1\right)$$

where δ is a normalization parameter that was calculated over the training set. *d* is the degree of the node that is getting the message. Then, we generalize this scaler as follows:

(15) $$S\left(d,\alpha \right)={\left(\begin{array}{c} \frac{log(d+1)}{\delta } \end{array}\right)}^{\alpha },d>0,-1⩽\alpha ⩽1$$

where α is the value of a variable parameter that can be negative for attenuation, positive for amplification, or zero for complete lack of scaling. Other definitions of S(d) can be used, such as a linear scaling definition, as long as the function is injective for S(d) greater than zero.

The principal neighborhood aggregation function is created by combining the aggregators and scalers described in the preceding sections (PNA). As detailed in following equation, we employed four neighbor-aggregations with three degree-scalers each to evaluate this general and flexible architecture.

(16) $$a_{v}^{\left(l\right)}=\underset{scalers}{\underset{︸}{\left[\begin{array}{c} I\\ S(\tilde{D},\alpha =1)\\ S(\tilde{D},\alpha =-1) \end{array}\right]}}⊗\underset{aggregators}{\underset{︸}{\left[\begin{array}{c} \mu \\ \sigma \\ max\\ min \end{array}\right]}}$$

where mean (μ), standard deviation (σ), max, and min are the aggregators defined in [52]; scalers are defined in Equation (15); and ⊗ is the product.

*M* convolutions were used for the experiments, and then three fully-connected layers were used to label nodes. This architecture, shown in Figure 5, was used for the experiments. After each layer’s update function, gated recurrent units (GRUs) [53] were added to each layer to help it run faster. Their ability to keep information from previous layers worked well when we added more convolutional layers *M*.

## 4. Experiments

### 4.1. Dataset

**Figure 6:**
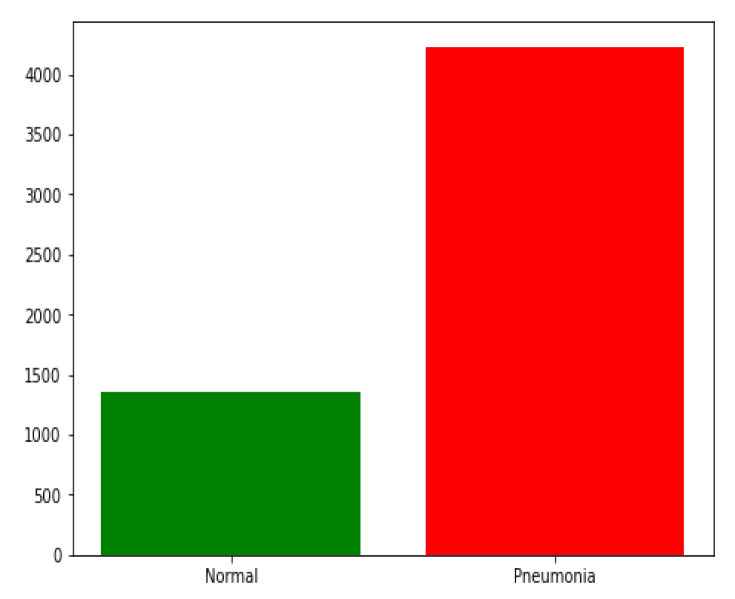
The training set distribution of image classes showing the disparity in data used for training.

In this research, we used chest X-ray images from Kaggle [54]. The dataset is available online. The dataset contains 5856 chest X-ray images in the JPEG format of children under six years old that were captured during routine clinical care of patients. As shown in Table 2, the training set contained 4232 images divided between 3883 images classified as depicting pneumonia and 1349 images classified as normal. It should be noticed the training set is imbalanced, as shown in Figure 6. The model was evaluated with 390 images classified as depicting pneumonia and 234 images classified as normal. Examples of healthy chest X-ray images and pneumonia infected chest X-ray images are shown in Figure 1 and Figure 2, respectively.

### 4.2. Experimental Settings and Evaluation Criteria

For the experiment, we used a consistent setup. We trained the model for 70 epochs with the Adam optimizer with an initial learning rate of $5\times 10^{-4}$ and weight decay of $10^{-6}$. The mini-batch sizes were set to be 32 and we used add as an aggregation operator. To reduce over-fitting, we utilized dropout with a rate of 0.6 for embeddings and used a rate of 0.3 for aggregation module outputs. We set the number of multi-head to be 4. We chose the image size to be 150×150×3. All our code was implemented in Pytorch [55].

#### Evaluation Criteria

We tested a number of models on the test dataset for a fair comparison. For model evaluation, we used four performance metrics: accuracy, precision, recall, and F1 score.

1. The accuracy metric is the ratio of the number correctly predicted images to the total number of images examined.
  (17) $$Accuracy=\frac{\left(True\;Positive+True\;Negative\right)}{total\;number\;of\;images}$$
2. The precision metric is the ratio of the number correctly predicted pneumonia images to the sum of the number correctly predicted pneumonia images and the number of normal images incorrectly identified as pneumonia images.
  (18) $$Precision=\frac{True\;Positive}{True\;Positive+False\;Positive}$$
3. The recall metric is the ratio of the number of correctly predicted pneumonia images to the sum of the number correctly predicted pneumonia images and the number of pneumonia images incorrectly identified as normal.
  (19) $$Recall=\frac{\left(True\;positive\right)}{True\;positive+False\;Negative}$$
4. F1 score is the weighted harmonic mean of recall and precision.
  (20) $$F1\;score=2\times \frac{\left(Recall\times Precision\right)}{Recall+Precision}$$

### 4.3. Results and Discussion

**Figure 7:**
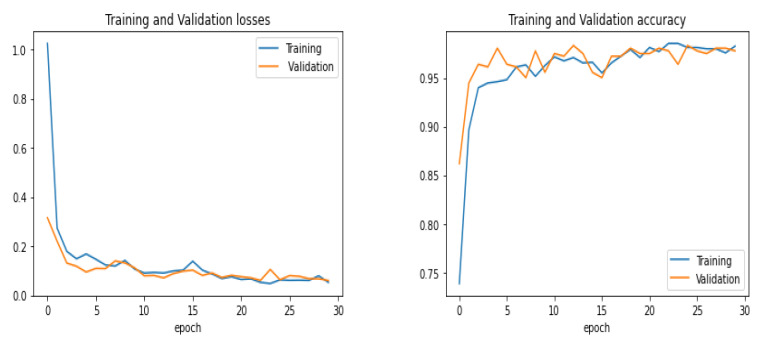
Training and validation performance.

**Figure 8:**
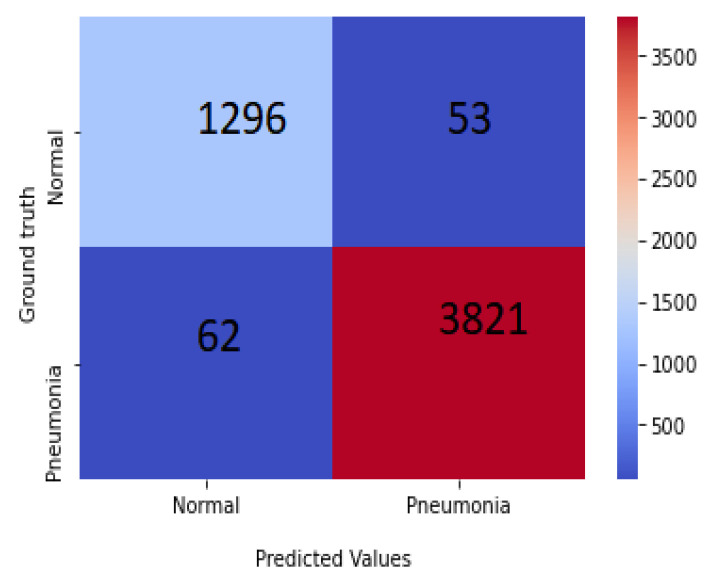
Confusion matrix of PNA-GCN on test data.

This section represents the experimental results achieved for the considered X-ray images with chosen dimensions. The equivalent convergence for accuracy and the loss function of the outcomes achieved from various convolution layers for training and validation process are shown in Figure 7, and the confusion matrix is shown in Figure 8. The merits of our method were further verified by comparing it with other algorithms from the literature, including those of Kermany et al. [54] and Lahsaini et al. [56]. The outcomes shown in Table 3 substantiate that the results achieved with the proposed method were better result compared to the other methods on the chosen dataset.

### 4.4. Ablation Study

#### 4.4.1. Input Size

**Figure 9:**
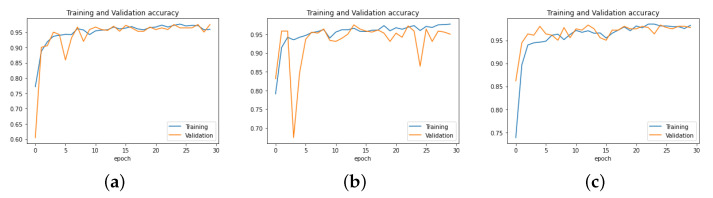
Performance of PNA-GCN with different data sizes. (**a**) Data size = 300 × 300 × 3. (**b**) Data size = 250 × 250 × 3. (**c**) Data size = 150 × 150 × 3.

The dimensions of processed images were fixed. To evaluate the validation performance of our model, we resized the chest X-ray images at 300 × 300 × 3, 250 × 250 × 3, and 150 × 150 × 3, respectively, and we trained them for 30 epochs each. The results obtained showed that the shape of the 150 × 150 image gave a better result than the others, as the model achieved higher validation accuracy with it. The experimental results are presented in Figure 9.

#### 4.4.2. The Influence of Batch Size N

**Figure 10:**
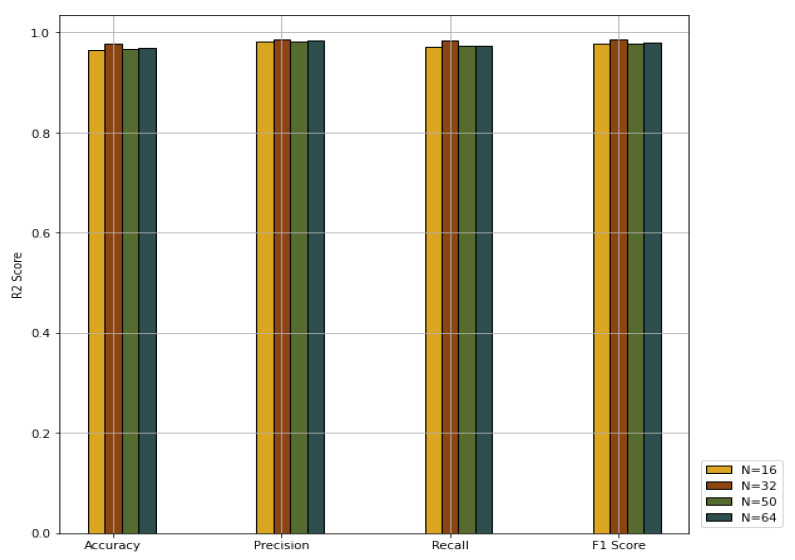
Performance comparison with different values for batch size.

We explored the effects of different values for the batch size *N* on the PNA-GCN performance. We chose 16, 32, 50, and 64, respectively. The experiment results plotted in Figure 10 show that PNA-GCN performs best with N=32, alleviating the memory problem during training.

#### 4.4.3. Effectiveness of the Multi-Head Attention

Rather than using the attention function just once, we use a multi-head attention layer, as [58] showed that the learning process can be stabilized using multiple independent attention operators. We collected the number *M* of multi-total heads within the set (1, 2, 4, 6, 8) to show the effectiveness of the multi-head attention. The experimental results plotted in Table 4 show that PNA-GCN performs best with the number of multi-heads being 4.

### 4.5. Influence of the Number of Aggregators

**Figure 11:**
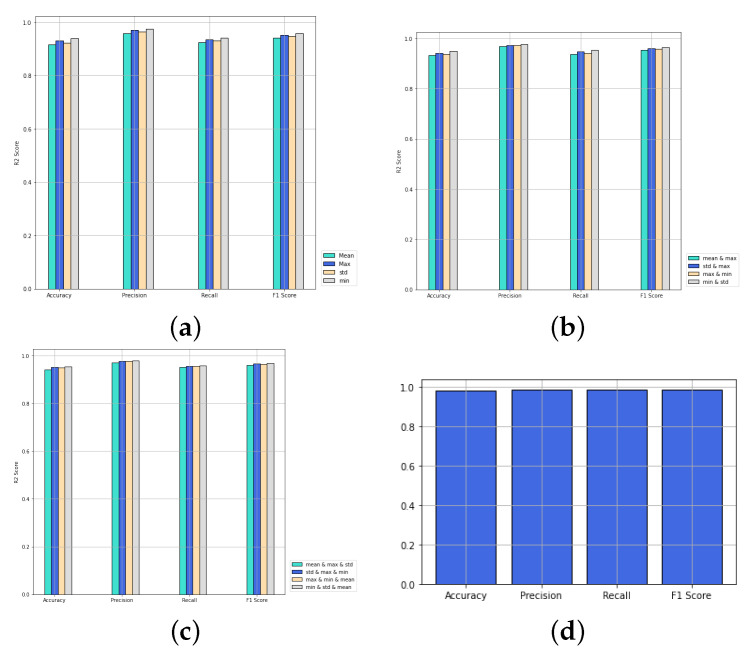
Performance of PNA-GCN with variable numbers of aggregators. (**a**) One aggregator; (**b**) two aggregators; (**c**) three aggregators; (**d**) four aggregators.

We compare the performance of PNA-GCN when making use of multiple aggregators along with degree-based scalers with single aggregators, as shown in Figure 11. PNA-GCN performs better with multiple aggregators. Specifically, PNA-GCN performs well with a combination of mean aggregation, standard deviation aggregation, max aggregation, and min aggregation. That is because the model can benefit from the strengths of each of them.

## 5. Conclusions

In this work, we proposed PNA-GCN, a principal neighborhood aggregation-based graph convolutional network for detecting pneumonia. The proposed model was developed taking into account the criteria of parameters and the time cost and memory cost of the training step, in contrast to other approaches that are based on transfer learning or use a more complex architecture. In this work, binary classification is executed and four different evaluation metrics have been used to evaluate the proposed method. The experimental results confirmed that the PNA-GCN achieved an improved outcome compared with other related approaches existing in the literature.

## Figures and Tables

**Table 1:** The settings for the image augmentation process.

Methods	Setting
Rotation range	30
Height shift	0.10
Width shift	0.10
Rescale	(1/255)
Zoom range	0.20
Shear range	0.20
Horizontal flip	True

**Table 2:** Dataset description.

	Pneumonia Infected Chest X-ray Images	Healthy Chest X-ray Images
Training set	3100	1073
Validation set	775	628
Test set	390	234

**Table 3:** Comparison of results.

	Accuracy	Precision	Recall	F1 Score
Kermany et al. [54]	92.8%	90.1%	93.2%	-
Lahsaini et al. [56]	93.24%	91.4%	95%	92.96%
Arunmozhi et al. [57]	97.65%	96.71%	98.32%	97.86%
PNA-GCN (Ours)	97.79%	98.63%	98.40%	98.51%

**Table 4:** Results comparison with different values for multi-head attention.

	M = 1	M = 2	M = 4	M = 8
Accuracy	96.12%	96.59%	97.79%	96.90%
Precision	97.99%	98.30%	98.63%	98.41%
Recall	96.75%	97.08%	98.40%	97.39%
F1 score	97.36%	97.69%	98.51%	97.90%

## Data Availability

Data is contained within the article.

## Abbreviations

The following abbreviations are used in this manuscript:

PNA	principal neighborhood aggregation
GCN	graph convolutional network
GRUs	gated recurrent units
GNNs	graph neural network
CNN	convolutional neural network
WHO	world health organization
